# The Dangerous Telephone-Box

**Published:** 1920-06-05

**Authors:** 


### THE DANGEROUS TELEPHONE-BOX.

WITH the threat of influenza upon us our Ministry of Health, with commendable zeal, has introduced a number of valuable prophylactic and precautionary measures. We would, however, humbly direct their attention to the public telephone-boxes in London.

The typical London telephone-box, such as is found in tube stations, post offices, and shops, is in height about nine feet and in width and depth about four. Ventilation of these "boxes" is appallingly insufficient: we would recommend the Ministry to take samples of the atmosphere at the end of six minutes' habitation of the "box" by an adult. We are sure that serious microbic concentration would be found if such samples were submitted to bacteriological analysis.

From the æsthetic point of view this lack of ventilation leaves much to be desired, for telephone-boxes are scarce in this great city, and happy is the lot of him who finds a public 'phone not in use when he wants it. Usually the "box" is inhabited, and at the busy time of the day as often as not several people are waiting patiently or impatiently to be "next man in." Some people smoke (!) whilst telephoning, or peradventure a scented female has preceded you, in which case you have to combat the unnatural perfumes of the Orient in addition to the physiological and probably congenital idiocy of the average telephone clerk! Then again, and we do not wish to labour the point, the telephone-boxes are used by unhealthy people and by those suffering from colds, coughs, incipient influenza, pulmonary tubercle, and infectious fevers. Of course, such unfortunate people always will use the telephone if they wish to, for the simple reason that there is nothing to prevent them: but we do know that if more commodious and better-ventilated boxes were provided the spread of infectious disease would be rendered more difficult.

The septic condition of the telephone itself at the busy time of the day is a disgrace to this City, and warrants the immediate attention of the health authority. All men have seen the moist or even wet state of the speaking-tube, and experienced the warm and greasy condition of the receiver. We should like an experienced bacteriologist to take cultures from a dozen of these instruments in the City of London area, and to report his findings to the medical profession and to the public at large.

We are not striving after the impossible, but only wish to bring before the authorities the disgraceful state of affairs we have endeavoured to describe, and which the "man in the street" is thoroughly acquainted with. The remedy is a simple one. Large, well-ventilated boxes should be provided, and their interior cleansed with a germicide at least twice a day, and some form of antiseptic paper cover should be provided for mouth- and ear-piece of the instrument.

We realise fully that such measures would be expensive, but the telephones are the property of the nation, and in the interests of political and social economy we can perceive no extravagance, but a very real economy, in the expenditure of any money devoted to such a laudable object.

